# Acute Esophageal Necrosis Secondary to Cardiogenic Shock: A Case Report and Review of the Literature

**DOI:** 10.7759/cureus.109736

**Published:** 2026-05-27

**Authors:** Magda K López Saldaña, Miguel A Galindo López, Sussan I Méndez Ynostroza, Francisco Rivera Martínez, Claudia P Contreras Saenz

**Affiliations:** 1 Internal Medicine, Centenario Hospital Miguel Hidalgo, Aguascalientes, MEX

**Keywords:** acute esophageal necrosis, black esophagus, cardiogenic shock, ischemic esophagitis, upper gastrointestinal bleeding

## Abstract

Acute esophageal necrosis (AEN), also known as "black esophagus," is an uncommon clinical entity characterized by diffuse, circumferential, dark pigmentation of the esophageal mucosa. Its pathogenesis is explained by a "two-hit" mechanism involving systemic hypoperfusion and local chemical injury from gastric acid. We present the case of a 78-year-old man with a history of hypertension, diabetes, and a recent anteroseptal myocardial infarction complicated by cardiogenic shock, requiring triple stent placement and vasopressor support. One week after discharge, the patient was readmitted due to hematemesis and hemodynamic instability. Upper gastrointestinal endoscopy revealed extensive necrotic tissue in the distal third of the esophagus, ending abruptly at the gastroesophageal junction. A thoracoabdominal computed tomography scan confirmed esophageal wall thickening but ruled out perforation. Management was strictly conservative, consisting of total parenteral nutrition for esophageal rest, aggressive acid suppression with intravenous proton pump inhibitors, and optimization of cardiac output. A follow-up endoscopy performed seven days later showed complete resolution of the necrotic lesions. Although AEN carries a high overall mortality rate, death is usually attributed to underlying comorbidities rather than esophageal complications. This case highlights that prompt stabilization of the triggering hemodynamic event, combined with conservative medical therapy, allows for rapid mucosal recovery and prevents the need for invasive surgical intervention.

## Introduction

Acute esophageal necrosis (AEN), also known as acute necrotizing esophagitis, black esophagus, or Gurvits syndrome [1,2], is a rare and potentially devastating medical condition that presents clinically as an unusual cause of upper gastrointestinal bleeding [1,3,4]. It is strictly defined by its endoscopic findings, which reveal a black, diffuse, and circumferential discoloration of the esophageal mucosa, indicating extensive tissue necrosis [1,4].

Black esophagus is an exceedingly rare clinical entity. A retrospective series of upper gastrointestinal endoscopies reports an extremely low incidence, ranging from 0.01% to 0.29% [1]. In autopsy series, the reported prevalence is 0%-2% [2,5]. However, its true frequency is likely underestimated, as esophageal mucosal damage can heal rapidly or present subclinically [5,6]. AEN is reported to be responsible for up to 6% of cases of upper gastrointestinal bleeding [5,7]. It typically occurs in older patients with a mean age of 67 years [2,4], and predominantly in male patients. Other important factors include a history of cardiovascular disease, hemodynamic compromise including shock, gastric outlet obstruction, alcohol abuse, use of nonsteroidal anti-inflammatory drugs, diabetes mellitus, malnutrition and hypoproteinemia, malignancies, renal disease, hypoxemia, and a hypercoagulable state, among others [4].

## Case presentation

We present the case of a 78-year-old unemployed male patient with a technical educational background. His medical history includes arterial hypertension and type 2 diabetes mellitus, treated with enalapril, metformin, and glibenclamide, with a recent glycated hemoglobin level of 6.4%. He also has a history of ischemic stroke and benign prostatic hyperplasia treated with tamsulosin.

In late November 2024, the patient presented with an anteroseptal ST-elevation myocardial infarction (Global Registry of Acute Coronary Events 174, thrombolysis in myocardial infarction flow 11, Killip-Kimball II). Diagnostic angiography revealed a dominant right coronary artery (RCA) system with multivessel obstructive disease: a short 40% ostial lesion in the left main coronary artery, a long and eccentric 95% stenosis in the proximal third of the left anterior descending (LAD) artery, and a 50% ostial-proximal lesion in the circumflex (left circumflex) artery. The dominant RCA exhibited diffuse lesions throughout its course, including an 80% proximal stenosis and a critical subocclusive lesion in the proximal segment of the posterior descending artery (PDA), which originated as a terminal branch of the RCA (Figure 1).

**Figure 1:**
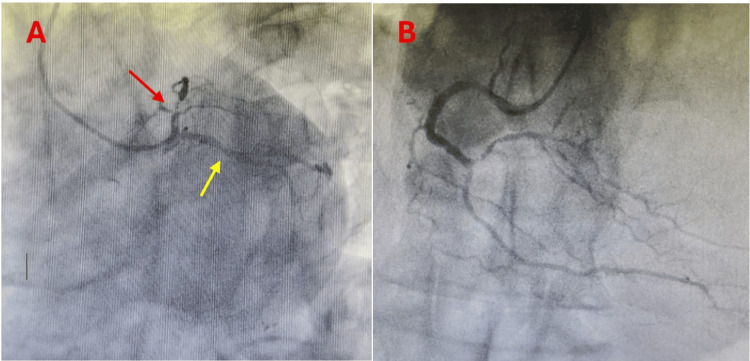
Pretreatment coronary angiography (A) Left coronary angiogram in the LAO caudal projection. The angiogram reveals a short 40% ostial lesion in the left main coronary artery. A long, eccentric, and critical 95% stenosis is clearly identified in the proximal third of the LAD artery (red arrow). Additionally, the circumflex (LCx) artery demonstrates a 50% lesion in its ostial-proximal segment (yellow arrow). (B) Right coronary angiogram in the LAO cranial projection. The angiogram demonstrates a dominant right coronary artery with severe, diffuse atherosclerotic disease throughout its course, significantly compromising distal blood flow prior to percutaneous intervention LAO: left anterior oblique; LAD: left anterior descending; LCx: left circumflex

During the procedure, the clinical course was complicated by acute intraprocedural cardiogenic shock and complete atrioventricular block, which required emergent temporary transvenous pacemaker placement and increased vasopressor support. Although current clinical guidelines give a Class III recommendation against routine immediate multivessel percutaneous coronary intervention (PCI) in cardiogenic shock, this patient experienced an acute, refractory electrical and circulatory collapse on the table. As a life-saving bail-out strategy to restore global myocardial perfusion, emergency multivessel intervention was successfully performed on three distinct coronary segments. A drug-eluting stent (DES) (Biomatrix Alpha, 3.0 x 24 mm; Biosensors International, Singapore) was deployed in the proximal LAD. Additionally, a DES (Biomatrix Alpha, 2.25 x 14 mm) was placed in the PDA, followed by a third DES (Biomatrix Alpha, 3.0 x 29 mm) implanted in the proximal RCA, achieving an optimal final distal TIMI 3 flow in both systems. The circumflex artery was not intervened due to the noncritical nature of its lesion (Figure 2).

**Figure 2:**
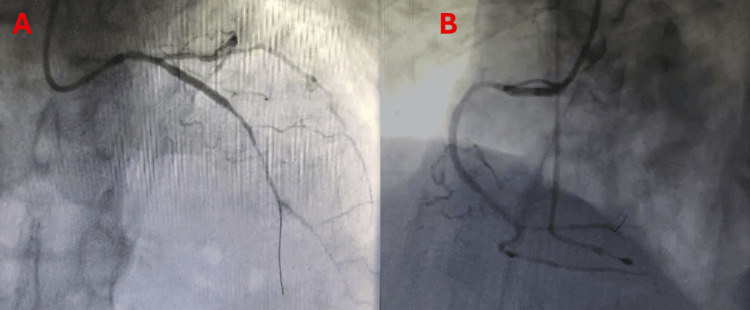
Posttreatment coronary angiography (A) Left coronary angiogram in the RAO cranial projection. The image demonstrates the final result following successful percutaneous coronary intervention of the LAD artery. The previously identified critical proximal stenosis has been completely resolved after the deployment of a drug-eluting stent, achieving optimal restoration of distal blood flow. (B) Right coronary angiogram in the LAO projection. The angiogram shows the final result after successful percutaneous coronary intervention of the dominant RCA. Optimal distal blood flow (TIMI 3) has been completely restored following the deployment of drug-eluting stents in the proximal RCA and the posterior descending artery RAO: right anterior oblique; LAD: left anterior descending; LAO: left anterior oblique; RCA: right coronary artery; TIMI 3: thrombolysis in myocardial infarction flow 3

A postprocedure transthoracic echocardiogram reported a left ventricular ejection fraction (LVEF) of 45%, a cardiac output of 3.9 L/minute, and an intracavitary thrombus in the left ventricle measuring 16 x 15 mm (Figure 3).

**Figure 3:**
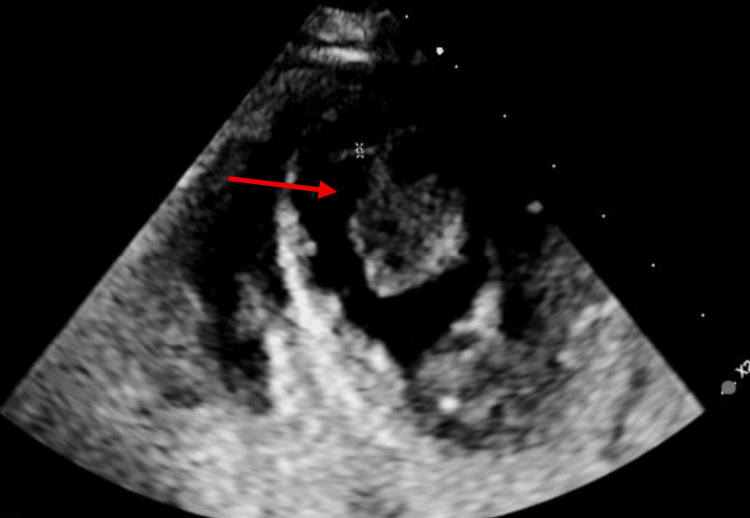
Transthoracic echocardiogram Off-axis apical view focusing on the left and right ventricles. The red arrow points to the persistent apical intracavitary thrombus in the left ventricle. No atrial chambers are visualized in this specific plane

During that hospitalization, the patient required support with norepinephrine and dopamine, which were subsequently and successfully weaned off. Additionally, during his stay, the patient presented a decrease in hemoglobin due to erosive gastropathy, which was treated with dual hemostatic therapy using epinephrine and bipolar coagulation (Figure 4). He also developed paroxysmal atrial fibrillation and flutter. He was eventually discharged on apixaban, clopidogrel, aspirin, atorvastatin, dapagliflozin, spironolactone, and furosemide.

**Figure 4:**
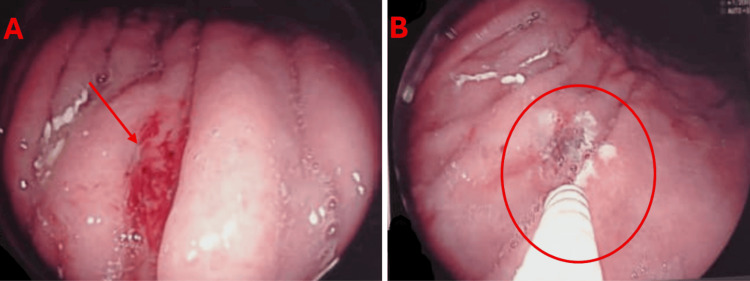
Endoscopic view of the gastric mucosa (A) Erythematous mucosa with linear erosions on the gastric folds (red arrow). (B) Instrumental maneuver on friable mucosa showing punctate bleeding (red circle)

In December 2024, the patient presented with hematemesis and respiratory distress, leading to readmission to the emergency department two days later. Upon admission, he was hemodynamically unstable, with a blood pressure of 89/59 mmHg, a heart rate of 117 bpm, and an oxygen desaturation of 81%. This severe hypotension was attributed to a mixed cardiogenic and hypovolemic etiology, triggered by acute gastrointestinal bleeding in the setting of his recent myocardial infarction and reduced cardiac reserve.

Physical examination revealed pallor, dehydration of the oral mucosa, and traces of blood in the oral cavity. Persistent arterial hypotension and tachycardia were documented. The abdomen was nontender to palpation with no signs of peritoneal irritation, and bowel sounds were present.

An emergency endoscopy was performed. During the procedure, the patient developed severe hypotension with a blood pressure of 64/32 mmHg (mean arterial pressure 42 mmHg), requiring the initiation of a norepinephrine infusion. The endoscopic report revealed that, starting at 26 cm from the incisors, the entire circumference of the esophageal mucosa was covered with fibrinous exudates and areas of interstitial hemorrhage. The distal third exhibited black tissue compatible with necrosis, which terminated abruptly at the gastroesophageal junction at 39 cm (Figure 5). In the stomach, a sparse accumulation of coffee-ground fluid with remnants of old blood was observed, and the mucosa showed irregular erythema and petechial subepithelial punctate lesions, leading to the diagnosis of cardiogenic black esophagus.

**Figure 5:**
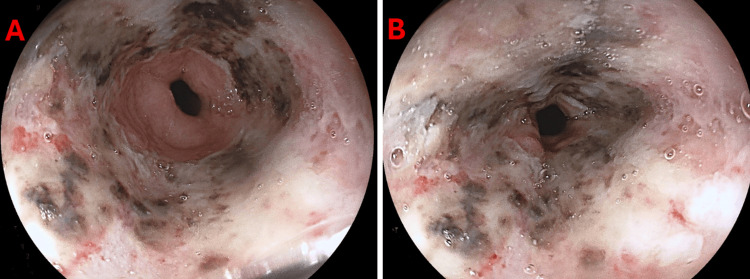
Acute esophageal necrosis Circumferential view of the esophagus and esophagogastric junction. (A) Esophageal mucosa with the presence of extensive and circumferential necrosis affecting the wall, extending toward the gastric folds at the esophagogastric junction. (B) Black-brownish adherent material and pseudomembranes are observed with areas of ulceration, resulting in a partially reduced esophageal lumen

Initial laboratory tests reported leukocytosis of 20.23 x 10³/µL (reference range: 4.5-11.00 x 10³/µL), moderate anemia with a hemoglobin level of 9.3 g/dL (reference range: 12.0-15.0 g/dL), and reactive thrombocytosis of 630 x 10³/µL (reference range: 150-450 x 10³/µL). Blood and urine cultures were drawn upon admission and subsequently returned negative, ruling out an infectious process and confirming the leukocytosis as a systemic inflammatory response to acute tissue necrosis.

A dual-contrast thoracoabdominal computed tomography (CT) scan was requested, which showed increased parietal thickness of the lower third of the esophagus (5 mm) with no evidence of contrast extravasation or extravisceral air, effectively ruling out esophageal perforation (Figure 6).

**Figure 6:**
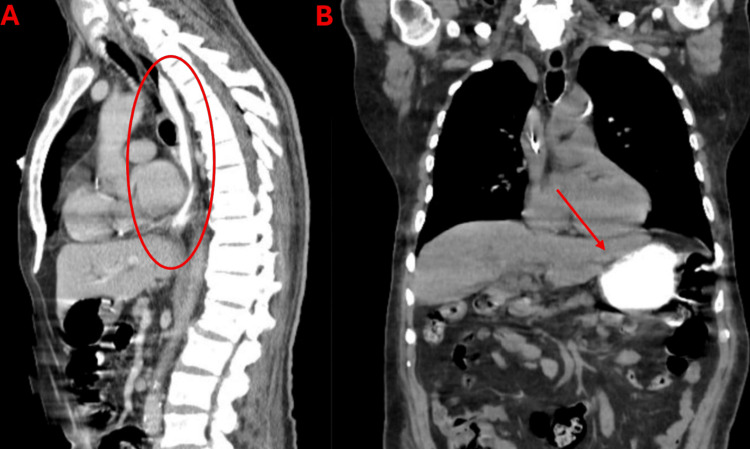
Double contrast chest and abdominal CT scan (A) Sagittal projection in which a red oval highlights the thickening of the esophageal wall, with no evidence of contrast extravasation, mediastinal collections, or appreciable pneumothorax. (B) Coronal projection where the red arrow points to the gastric chamber with adequate contrast passage and no apparent leaks

During hospitalization, the patient received conservative management as he did not present complications inherent to esophageal necrosis. An evaluation by the general surgery service was requested, which confirmed that surgical intervention was not required. The patient was kept nil per os for five days, and total parenteral nutrition (Kabiven 1400 kcal) was initiated, with no electrolyte imbalances. Intravenous proton pump inhibitor therapy was administered (omeprazole 40 mg every 12 hours), and heart failure management was continued. Following a favorable clinical course and achieving hemodynamic stability without the need for vasopressors, a follow-up endoscopy was performed in December 2024, showing significant improvement of the esophageal mucosa with no evidence of necrotic lesions (Figure 7). The patient tolerated progression to an oral diet and was discharged due to clinical improvement.

**Figure 7:**
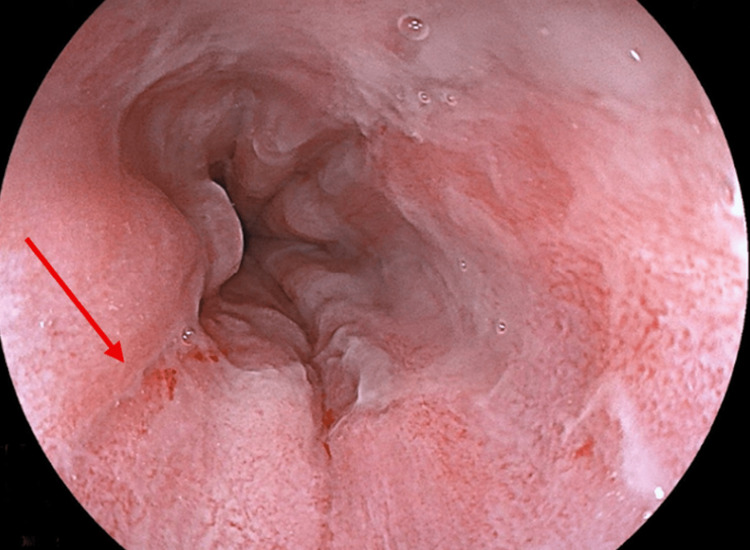
Follow-up endoscopy after conservative treatment Endoscopic view of the esophagus showing significant mucosal improvement and healing compared to the previous study, with pink mucosa, partial reepithelialization, and complete resolution of the necrotic plaques. Mild areas of erythema persist, and the red arrow indicates residual mucosal erosions without any evidence of remaining necrotic material

A follow-up transthoracic echocardiogram performed prior to discharge demonstrated an LVEF of 41%, a cardiac index of 3.1 L/minute/m^2^, and signs of elevated filling pressures (estimated pulmonary capillary wedge pressure of 35 mmHg). The apical left ventricular intracavitary thrombus persisted through the end of the hospital stay. Consequently, long-term oral anticoagulation with apixaban was maintained at discharge to target thrombus resolution during outpatient follow-up.

## Discussion

The pathogenesis of AEN is explained by the "two-hit" model [3,8]. In this hypothesis, a weakened esophageal mucosa suffers severe acute damage [3]. The initial hit involves an acute hypoperfusion event that compromises local mucosal defense mechanisms, rendering the esophagus highly vulnerable to ischemic injury [8]. The second hit occurs when a direct and severe injury is caused by the massive reflux of gastric acid and pepsin onto a mucosa that is already ischemic and unprotected [8].

Anatomically, the involvement in our patient predominated in the distal third, consistent with the description of this zone as a vascular watershed area [1]. The esophageal blood supply is segmental. The proximal esophagus is supplied by the inferior thyroid arteries; the middle esophagus by the bronchial arteries and direct aortic branches; and the distal esophagus depends almost exclusively on the left gastric artery and the left inferior phrenic artery [5,6,9,10]. Unlike the upper and middle thirds, which have a robust collateral network, the distal esophagus is extremely vulnerable to drastic drops in systemic perfusion pressure [2,5].

In our patient, cardiogenic shock secondary to an anteroseptal myocardial infarction acted as the initial ischemic trigger. Cardiogenic shock severely compromises systemic circulation, precipitating a downward hemodynamic spiral. The initial event is cardiac pump failure with a consequent drop in cardiac output and a hypotensive state [11]. The main characteristic of cardiogenic shock is peripheral vasoconstriction as the body's response to compensate for pressure failure and divert blood to vital organs [11]. The increase in afterload due to vasoconstriction generates an overload of work on an already severely damaged myocardium [11]. The failure to maintain an adequate stroke volume and ineffective circulatory compensation critically decrease blood flow to peripheral tissues, generating generalized tissue hypoperfusion and ischemic organ damage [11].

Cardiogenic shock precipitates AEN by inducing systemic hemodynamic collapse. The primary reduction in cardiac output and myocardial inotropy leads to a generalized state of hypoperfusion [1]. To sustain systemic blood pressure, the body initiates a compensatory, intense peripheral and splanchnic vasoconstriction [11]. At the microcirculatory level, this response critically reduces blood flow to the capillary networks of the esophageal mucosa. This ischemic insult predominantly affects the distal third of the esophagus, which functions as a vascular watershed zone; its lower density of terminal vessels and lack of robust collateral networks render it highly vulnerable to sudden drops in blood flow [1,12]. Additionally, adequate microcirculation within the lamina propria and submucosa is essential to sustain local buffering systems and cellular repair mechanisms. Ischemia inactivates these defenses, leaving the mucosa entirely vulnerable to secondary chemical injury [1,5]. Furthermore, some reports suggest that low-flow states can induce localized esophageal spasms, which further compromise microscopic perfusion and directly contribute to transmural tissue necrosis [12].

Regarding the demographic profile, there is a clear predilection for the male sex, representing around 70%-73% of all cases [1,5,6,9]. The incidence increases with age, peaking between the sixth and seventh decades of life [5,6,9]. Close to 78% of diagnosed patients are 50 years or older [8].

It is noteworthy that our patient presented multiple risk factors for AEN, such as diabetes and a history of ischemic heart disease. AEN is predominantly reported in individuals with general health deterioration, malnutrition, and multiple underlying diseases [1]. Common comorbidities include diabetes mellitus (36%-38%) [1,9], arterial hypertension (37%), heart failure (7%), and coronary artery disease (12%), which predispose patients to ischemic episodes [1,4]. AEN is almost always precipitated by a severe acute medical condition, such as hemodynamic shock [1,9].

Our patient presented gastrointestinal bleeding, objectively evidenced by hematemesis, which is typical, as 85%-90% of AEN patients manifest upper gastrointestinal bleeding [5,9]. He also presented with shock requiring vasoactive amines, aligning with the literature stating that clinical signs reveal severe hypotension and hemodynamic instability [3,6,9,10]. It is important to clarify that this shock state is not solely a consequence of hypovolemia from the gastrointestinal bleeding; rather, it represents a unique, mixed mechanism inherent to AEN. The widespread ischemic tissue necrosis of the esophagus triggers a profound systemic inflammatory response that causes severe distributive vasodilation, which, in this case, critically aggravated the patient’s preexisting cardiogenic and hypovolemic vulnerability.

This pathology features a well-established endoscopic classification system used to monitor disease progression and detail the distinct phases of black esophagus. Stage 0, or the prenecrotic esophagus, involves viable tissue without evidence of necrosis. Stage 1 represents the acute phase, or classic black esophagus, which is the defining stage of the condition; it is characterized by a friable, diffuse, and circumferential necrotic mucosa with occasional yellowish exudates that typically originate at the esophagogastric junction and extend variably upward into the proximal esophagus. This was the exact clinical stage identified in our patient upon admission. Subsequently, Stage 2 reflects the healing phase-often described as having a chessboard appearance, distinguished by residual necrotic patches alternating with thick, white exudates that cover a pink, friable underlying mucosa. Finally, Stage 3 is the recovery phase, where the esophageal mucosa completely restores its normal endoscopic appearance; our patient successfully demonstrated this advanced healing process during the follow-up endoscopy after conservative management, showing extensive reepithelialization with only residual mucosal erosions remaining [5].

The general prognosis for patients diagnosed with black esophagus is poor. Clinical series show a high overall mortality rate ranging from 29.9% to 32%. It is important to note that patients presenting with AEN who are in shock upon admission carry a dismal prognosis, with mortality rates reaching up to 50% [1,5,8,9]. It is crucial to highlight that this high lethality is rarely due directly to esophageal perforation. Most deaths result from general deterioration and underlying chronic diseases such as multiorgan failure or shock [1,2,6,10]. In contrast, the specific mortality directly attributable to local esophageal complications, such as perforation or mediastinitis, is reported at only 6% [1,6,10].

Currently, there is no specific curative therapy. Treatment is primarily supportive and conservative in approximately 75% of cases [1,4]. Supportive measures rely on hemodynamic resuscitation, bowel rest, and parenteral nutrition [1,5,8]. Surgical treatment is reserved only for complications like perforation or mediastinitis [1,5,6]. In this case, since the double-contrast CT ruled out perforation, conservative treatment was chosen. A critical point for the resolution of AEN in our patient was the resolution of the hemodynamic shock and the compensation of heart failure, aimed at restoring blood flow and halting necrosis [1]. Furthermore, intravenous proton-pump inhibitors were administered to block acid secretion, drastically reducing corrosive exposure [3]. The patient's successful discharge was due to the resolution of the primary cause (the shock state), reinforcing that mortality in this pathology is centered on the severity of the underlying systemic diseases [1,3,5].

## Conclusions

AEN is an infrequent clinical entity with a high mortality rate that is closely linked to the severity of underlying systemic pathologies. Early diagnosis through endoscopy is essential to differentiate it from other causes of upper gastrointestinal bleeding. In patients with low cardiac output states, such as cardiogenic shock, vigilant monitoring for gastrointestinal symptoms is crucial, given that the distal third of the esophagus represents a critical zone of ischemic vulnerability. This case highlights that therapeutic success does not depend on invasive surgical interventions in the absence of perforation, but rather on timely, aggressive conservative management. Optimization of hemodynamic status, esophageal rest supported by parenteral nutrition, and intensive acid suppression are the therapeutic pillars that facilitate rapid and complete mucosal recovery. Ultimately, this report reinforces that managing the triggering systemic cause remains the most effective measure to reverse ischemic esophageal damage.
